# Comparison of Psychiatric Service Utilization Prior, During, and After COVID-19 Lockdown: A Retrospective Cohort Study

**DOI:** 10.7759/cureus.33099

**Published:** 2022-12-29

**Authors:** Bashaer Jahlan, Imtinan Alsahafi, Eman Alblady, Rami Ahmad

**Affiliations:** 1 Department of Family Medicine, King Abdulaziz Medical City, Ministry of the National Guard - Health Affairs, King Abdullah International Medical Research Center, Jeddah, SAU; 2 Department of Psychiatry, King Abdulaziz Medical City, Ministry of the National Guard - Health Affairs, King Abdullah International Medical Research Center, Jeddah, SAU

**Keywords:** services, psychiatric disorder, mental health care, lockdown, covid-19

## Abstract

Background

COVID-19 pandemic represents a significant risk factor for developing, relapsing, or exacerbating pre-existing mental health conditions. This negative impact on mental health results in increasing demand for psychiatric services. This study aimed to explore the effects of COVID-19 pandemic lockdown on the utilization of mental health services in three periods - prior, during, and after the lockdown - compared to the matched weeks in the previous years 2018 and 2019.

Materials and Method

In this retrospective cohort, quantitative, single-center study, data were collected from electronic medical records, including all patients with referrals\consultations to the psychiatric section prior, during, and after COVID-19 lockdown.

Results

In total, 2,454 patients were either referred to psychiatric outpatient clinics or needed consultation as inpatients during the study periods. Only 2,326 patients were included in our study. The total number of inpatient consultations was 1,410, with a statistically significant increase during the lockdown (p-value<0.001) and post-lockdown (p<0.016) in comparison to previous years. A significant reduction in outpatient referrals was observed during the lockdown (p=0.005) and post-lockdown period. Psychiatric disorders were identified in most patients (N=1,599), representing 65%, 54%, and 74% of patients in pre-lockdown, lockdown, and post-lockdown periods compared to 71%, 71%, and 76%, respectively, in the previous years. A total of 821 patients manifested symptoms of depression, constituting the largest proportion among all reasons for referral\consultations. The number of patients referred for substance/alcohol use disorders during the lockdown increased compared to patients in the same period in 2019. Suicidal behavior was identified in 70 patients across all study periods, with the lowest number observed in 2020.

Conclusion

Our findings indicate that during the COVID-19 lockdown, a significant increase in inpatient psychiatric services utilization was observed. Outpatient psychiatric service utilizations were significantly reduced. Implementation of evidence-based policy and protocol to guide mental health challenges in future health emergencies is needed.

## Introduction

The World Health Organization (WHO) officially declared coronavirus disease 2019 (COVID-19) as a pandemic on March 11, 2020 [1]. Many governments worldwide have implemented protocols to limit the spread of COVID-19. In this context, Saudi ministries have adopted several measures and regulations in different sectors including travel restrictions, the closure of educational institutions and launching of virtual learning, the closure of commercial centers, and a partial lockdown imposed initially for a few hours, followed by complete lockdown in most Saudi cities from May 23 to June 27, 2020. The COVID-19 pandemic has affected the political, industrial, economic, educational, and health care systems globally and influenced every aspect of life.

WHO has recognized mental health as an integral part of the COVID-19 response protocol [2,3]. The pandemic represents a significant risk factor for developing, relapsing, or exacerbating pre-existing mental health conditions. Early in the pandemic, a study in the USA was conducted on people with no previous history of psychiatric disorders, which showed that more than one in four persons had experienced mental distress [4]. In a more recent systematic review, the rate of symptoms of anxiety, depression, post-traumatic stress disorder, psychological distress, and stress were dominating during COVID -19 pandemic among the general population in many countries [5]. This negative impact results in increasing demand for psychiatric services. In addition, hospital services are challenged by the pandemic, which leads to a decrease in the delivery of essential services to the population.

In Saudi Arabia, a study showed that 23.6% of 1,160 participants reported psychological distress; patients with pre-existing psychiatric disorders had a higher score on the Event Scale-Revised (IES-R) and the Depression, Anxiety, and Stress Scales (DASS-21) [6].

In Saudi Arabia, the impact of COVID-19 and the precautionary regulations on mental health service utilization is unknown. To our knowledge, no previous study has been conducted regarding this in our country. This study aimed to explore the effects of COVID-19 pandemic lockdown on the utilization of mental health services in three periods - prior, during, and after the lockdown - compared to the matched weeks in the previous years, 2018 and 2019.

## Materials and methods

This is a retrospective cohort, quantitative study conducted in King Abdulaziz Medical City in Jeddah, Saudi Arabia. Target population included any adult patient aged 18 years and above who have been referred to the psychiatric outpatient department or had psychiatric consultation during inpatient admission. The study aimed to compare the rate of referrals/admissions during three periods of time (prior, during, and after COVID-19 lockdown) over 39 weeks, which corresponds to 13 weeks before COVID-19 lockdown from (December 22, 2019, to March 22, 2020), 13 weeks during the lockdown from (March 23, 2020, to June 21, 2020), and 13 weeks after the lockdown, from (June 22, 2020, to September 21, 2020), in comparison to the matched weeks in the previous years of 2018 and 2019. Retrospective data were collected from Health Information System (HIS) electronic patients’ records (Best Care). Patients aged less than 18 years and with referral and consultations before and after the study periods were excluded.

Ethical approval was obtained from the Institutional Review Board (IRB) vide Letter No. IRBC /0417/21, Study number: RJ20/243/J, dated March 2, 2021, with a waiver of written consent since data were collected only from medical records of patients.

Retrospective data were collected using structured data form. The data collection form was tested for content validity by two independent researchers. A pilot study was conducted where assessment of the study design, evaluation of the methodology, determining the feasibility of data abstraction, highlighting the frequency of missing variables in patients’ medical records, and the adequacy of the coding scheme was performed.

Data were analyzed using IBM SPSS version 23 (IBM Corp., Armonk, NY, USA), A simple descriptive statistic was used to define the characteristics of the study variables through the form of counts and percentages for the categorical and nominal variables, while continuous variables were presented by mean and standard deviations. To establish a relationship between categorical variables, this study used the chi-square test. Lastly, a conventional p-value of <0.05 was the criterion to reject the null hypothesis.

## Results

General characteristics of the patients

Overall, 2,454 patients were either referred to psychiatric outpatient clinics or needed consultation as inpatients during the study periods. Only 2,326 patients met the inclusion criteria and were included in our study. Any missing demographic and medical data were labeled as unknown due to incomplete documentation in the electronic medical record. Of the 2,326 patients, 1,235 (53.1 %) were females and 1,091 (46.9%) were males. Age was categorized into four subgroups, starting from the age group of 18-34 years to the age group of older than 65 years. Most of the patients (29.2%, n = 680) were aged 65 years and older, while the least number of referrals and consultations was in patients aged 18-34 years (22.3%, n = 519). Most of the patients (48.2%) had a previous history of psychiatric disorder, with depression being the most reported diagnosis (43.1%), followed by anxiety (17.9%); 44.5% of patients had no relative known to have a psychiatric disorder, and 14.9% had at least one family member affected with a psychiatric disorder. The demographic and medical characteristics of all patients are shown in Tables 1, 2.

**Table 1:** Demographic characteristics of the patients with referral and consultation to the psychiatry section

Demographics characteristics		Count	%
Total		2326	100.0
Age	18-34 years	519	22.3
	35-49 years	571	24.5
	50-64 years	556	23.9
	65 years and above	680	29.2
Gender	Male	1091	46.9
	Female	1235	53.1
Educational level	Unknown level of education	1108	47.6
	Illiterate	307	13.2
	Primary and secondary education	502	21.5
	Higher level of education	409	17.6
Occupation	Unknown occupational history	642	27.60
	Unemployed\retired	1143	49.14
	Employed (soldier, others)\students	484	20.80
	Health care workers	57	2.45
Marital status	Unknown marital history	252	10.8
	Single	330	14.2
	Married	1418	61.0
	Divorced\widowed	326	14.0
Living place	Unknown living place	503	21.6
	Jeddah city	1315	56.5
	Makkah\Al Taif city	185	7.9
	Al Madinah city\others	210	9.0
Family history of psychiatric disease	Unknown family history	943	40.6
	Yes	353	15.0
	No	1034	44.5
Previous psychiatric diagnosis	Unknown previous psychiatric history	352	15.1
	Yes	1120	48.2
	No	854	36.7

**Table 2:** Medical characteristics of the patients with referral and consultation to the psychiatry section

Previous psychiatric diagnosis	Count	%
Total	1120	100.0
Unknown psychiatric diagnosis	142	12.7
Depression	483	43.1
Anxiety	201	17.9
Insomnia	37	3.3
Psychosis	99	8.8
Dementia	72	6.4
Substance use disorder	66	5.9
Bipolar disorder	55	4.9
Suicide	14	1.3
Other psychiatric diagnoses	148	13.2
Referral issuing place		
Emergency department	170	7.3
Inpatient service	1252	53.8
Outpatient clinic	506	21.8
Primary health care center	398	17.1
Urgency of referral and consultation		
Routine referral	2027	87.1
Urgent referral	179	7.7
Emergency referral	120	5.2

Inpatient consultations to the psychiatry department

The total number of inpatient consultations was 1,410, with a higher rate noticed in the three study periods of the year 2020. A significant increase in inpatient consultations was noticed within the lockdown and post-lockdown period relative to the control groups within the matching weeks of previous years (p<0.05). There were a total of 246, 279, and 277 consultations in pre-lockdown, lockdown, and post-lockdown periods, respectively, compared to 208, 177, and 223 in the previous years (Table 3).

**Table 3:** Comparison of referrals and consultations to the psychiatry department across study periods Note: Values in the same row and subtable not sharing the same subscript are significantly different at p<0.05 in the two-sided test of equality for column proportions. Cells with no subscript are not included in the test. Tests assume equal variances and are adjusted for all pairwise comparisons within a row of each innermost subtable using the Bonferroni correction. *Significant using the chi-square test at <0.05 level

Variables		Request Date						χ2	P-value
		December 22, 2018, to March 22, 2019 (control)	December 22, 2019, to March 22, 2020 (pre-lockdown)	March 23, 2019, to June 21, 2019 (control)	March 23, 2020, to June 21, 2020 (lockdown)	June 22, 2019, to September 21, 2019 (control)	June 22, 2020, to September 21, 2020 (post-lockdown)		
		Count	Count	Count	Count	Count	Count		
Site of referral	Consultation service	208	246	177	279	223	277	84.653	<0.001*
	Outpatient referral	192	190	141	69	181	143		

**Figure 1:**
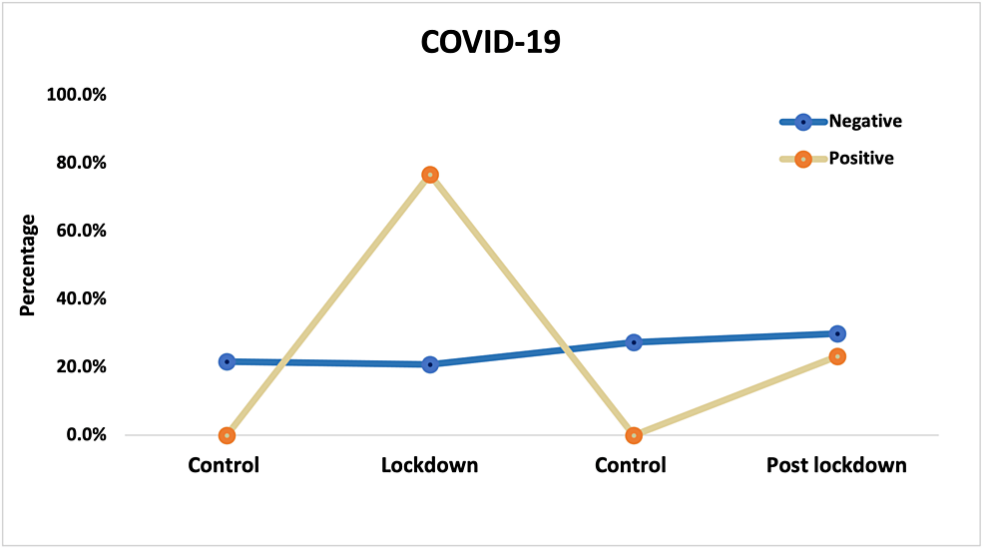
Linear correlation between the number of consultations and the number of COVID-19 cases

COVID-19 infection was reported in 109 patients out of 279 consultations during the lockdown period and in 33 patients out of 277 consultations in the post-lockdown period (Table 4, Figure 1).

**Table 4:** Reported COVID-19 infection among inpatients consultations *Significant using the chi-square test at <0.05 level

Variables		Total	COVID-19 Positive		P-value
			No	Yes	
Total		956	814	142	-
Request date	March 23, 2019, to June 21, 2019	177 (18.5%)	177 (21.7%)	0 (0.0%)	<0.001*
	March 23, 2020, to June 21, 2020 (lockdown)	279 (29.2%)	170 (20.9%)	109 (76.8%)	
	June 22, 2019, to September 21, 2019	223 (23.3%)	223 (27.4%)	0 (0.0%)	
	June 22, 2020, to September 21, 2020 (post-lockdown)	277 (29.0%)	244 (30.0%)	33 (23.2%)	

Outpatient referrals to the psychiatry department

The total number of outpatient referrals was 916. Referral number reduced from 190 in the pre-lockdown period to 69 during lockdown, with a slight increase in the post-lockdown period (N= 143, 15.6%). Compared to the control groups in previous year, a similar number of referrals is noticed in the pre-lockdown period (n=190) and control group (n=192) with significant reduction in lockdown period (p<0.001) and post-lockdown period (p<0.035) (Table 3).

Reasons for referrals and consultations to the psychiatry department

**Figure 2:**
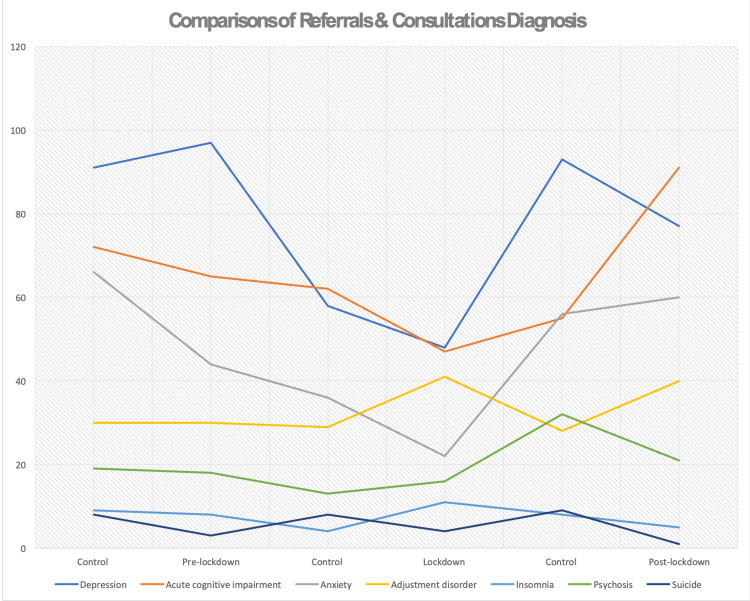
Comparison of referrals and consultation psychiatric diagnosis during pre-lockdown, lockdown, and post-lockdown.

The reported reasons for referral to mental health services were not mutually exclusive, as many referral letters stated multiple reasons for referral. Across all inpatients and outpatients, a total of 821 patients manifested symptoms of depression, constituting the largest proportion among all reasons. Higher numbers of patients were referred in 2019 in comparison to the pandemic year. The percentages were 38% (n= 164) in the pre-lockdown period, 24% (n=85) during lockdown, and 28% (n=116) in the post-lockdown period compared to 43% (n=170), 34% (n=109), and 44% (n=177) in the same periods in 2018/2019. The second frequent reason reported was anxiety, which varied across the periods from 13% (n=58) in the pre-lockdown period to 10% (n=36) during the lockdown and 11% (n=46) in the post-lockdown period, followed by agitation and other reasons including bipolar symptoms, behavioral changes, somatic symptoms, and personality disorder. A high number of patients were referred for the need of psychiatric assessment (31%, n=109) during the lockdown period compared to 2019 (2.2%, n=7). A total of 70 patients were referred due to suicidal behavior across all periods: 1.4% (n=6), 3% (n= 10), and 3% (n=12) in the pre-lockdown, lockdown, and post-lockdown period, respectively, compared to 3% (n=19), 5% (n=15), and 3.5% (n=14) in 2019/2018. The number of patients referred because of substance/alcohol use disorders during the lockdown increased to 2% (n=9) patients compared to 0% (n=0) patients in 2019. The rate of patients referred because they missed their follow-up visits was noticed to be higher in the post-lockdown period (19%, n=79) compared to 2019 (7%, n=29). For more illustration, see Figure 2 showing comparison of referrals and consultation psychiatric diagnosis during pre-lockdown, lockdown, and post-lockdown.

Outcome of referrals and consultations to the psychiatry department

Among all patients, a total of 420 patients failed to show up to their scheduled appointment. A total of 105 (24%) patients did not show up in the pre-lockdown period compared to 80 (20%) patients in the previous year. During the lockdown, 51 (14.7%) patients did not respond to virtual phone appointments and consultations compared to 59 (18.6%) patients in the previous year. In the post-lockdown period, 67 (16%) patients did not show up compared to 58 (14.3%) patients in the previous year. Psychiatric disorders were identified in most referred patients (N=1,599), representing 65%, 54%, and 74% of patients in pre-lockdown, lockdown, and post-lockdown periods, respectively, compared to 71%, 71%, and 76%, respectively, in the previous years. Notably, depression disorder was the most identified diagnosis, with 464 patients. During the lockdown, referrals with depression diagnosis (N=13) were significantly lower (p=0.005) with 13 patients compared to 32 patients in the previous year, as shown in Table 5. There were no other statistical differences in depression diagnosis, whether inpatient or outpatient referrals in different periods.

**Table 5:** Psychiatric disorder outcome across all study periods. Note: Values in the same row and subtable not sharing the same subscript are significantly different at p<0.05 in the two-sided test of equality for column proportions. Cells with no subscript are not included in the test. Tests assume equal variances and are adjusted for all pairwise comparisons within a row of each innermost subtable using the Bonferroni correction ^1^This category is not used in comparisons because its column proportion is equal to zero or one. *Significant using the chi-square test at <0.05 level

Variables		Request date						χ2	p-value
		December 22, 2018, to March 22, 2019 (control)	December 22, 2019, to March 22, 2020 (pre-lockdown))	March 23, 2019, to June 21, 2019 (control)	March 23, 2020, to June 21, 2020(lockdown))	June 22, 2019, to September 21, 2019 (control)	June 22, 2020, to September 21, 2020 (post-lockdown)		
		Count	Count	Count	Count	Count	Count		
Depression									
Site of referral	Consultation service	43	58	26	35	56	42	11.741	0.039*
	Outpatient referral	48	39	32	13	37	35		
Anxiety									
Site of referral	Consultation service	30	20	12	14	15	28	12.644	0.027*
	Outpatient referral	36	24	24	8	41	32		
Acute cognitive impairment									
Site of referral	Consultation service	71	65^1^	60	47^1^	54	91^1^	5.522	0.356
	Outpatient referral	1	0^1^	2	0^1^	1	0^1^		
Adjustment disorder									
Site of referral	Consultation service	26	28	28	41^1^	25	40^1^	10.792	0.056
	Outpatient referral	4	2	1	0^1^	3	0^1^		

Acute cognitive impairment was the second most common diagnosis identified in 392 patients during all study periods. A statistically significant increase (p=0.002) was observed only in the consultation services in the post-lockdown period, with a total of 91 patients in comparison to 51 patients in the previous year. Anxiety disorders were the third most common diagnosis in 284 patients. During the lockdown period, there was a significant reduction (p=0.005) in anxiety diagnosis among outpatients, with eight patients compared to 24 patients in the previous year. In the post-lockdown period, there was a significant increase (p=0.047) in consultation service, with 28 patients compared to 15 patients in the previous year. The fourth common diagnosis was adjustment disorder, with 198 patients being diagnosed with adjustment disorders with no statistical differences across all study periods. Some patients were transferred to another hospital with psychiatric admission services, with 0.7%, 0.6%, and 0.7% in pre-lockdown, lockdown, and post-lockdown periods, respectively, compared to 2.5%, 1.8%, and 1%, respectively, in previous years. No psychiatric illness was identified in 263 patients, with the most significant proportion (30%) during the lockdown compared to 9% in the previous year. Many patients needed medication prescriptions or adjustment, with 48%, 43%, and 66% in the pre-lockdown, lockdown, and post-lockdown periods, respectively compared to 59%, 54%, and 63%, respectively, in previous years.

## Discussion

This study is the first to assess the impact of the COVID-19 lockdown on psychiatric services utilization in Saudi Arabia. The total reduction in outpatient referrals for the year 2020 was observed, with a significant reduction (p <0.05) in both lockdown and post-lockdown periods compared to the previous year. The reduced referrals should be interpreted with some caution; however, similar results were reported in Denmark, where a 40% decline in referrals to the mental health service was noted during the COVID-19 pandemic [7], and in a retrospective study conducted in the United Kingdom, where a statistically significant decrease in referral to psychiatric utilities during COVID-19 pandemic [8]. The reduction in referrals can be attributed to multiple reasons including restrictions in medical services, shortage of medical staff, and patient avoidance of health care facilities in fear of contracting COVID-19. The difference in results can be attributed to many causes including the methodological designs differences, and cultural and resilience factors.

A significant increase (p<0.05) was found in inpatient consultations in the lockdown and post-lockdown periods relative to the control groups. Overall, the highest number of consultations was observed during the lockdown period. This increase can be explained by the implementation of inpatient protocol in our institute where a psychiatric assessment was offered during the lockdown to all patients admitted with COVID-19 disease in response to the release of the World Health Assembly [9] and local health authorities' recommendations to reinforce protective mental health measures during public health emergencies. The results show that a total of 109 patients were referred for psychiatric assessment.

In addition, a slight increase in inpatient consultations was noted in the post-lockdown period (N=277). This increase might be attributed to the relapse of pre-existing mental health disorders, the development of new psychiatric conditions caused by COVID-19 disease-related consequences, loss of social support, and the difficult access to mental health services during the pandemic restrictions. Similar findings in Switzerland showed a significant increase in post-lockdown psychiatric emergency consultations compared to the lockdown period [10].

In contrast to our results, a study conducted in the Netherlands assessing the mental health changes of 1,519 participants over 10 weeks, including five weeks post-restrictions removal, reported that most of the participants remained in stable mental health status; furthermore, some reported a positive effect of COVID-19 on their well-being [11]. Different sampling techniques may cause the difference in results, methodological designs or can be due to differences in culture and resilience factors. In addition, our results represent a narrow spectrum of the general population in contrast to the results reported by Gijzen et al., which were based on the general population [11].

Psychiatric disorders were identified in most referred patients. Depression was the most frequently reported reason for outpatient referrals and inpatient consultations. It was observed to be the most common psychiatric diagnosis through all study periods with a significant reduction (p-value=0.005) during the lockdown in outpatient visits, followed by anxiety, which was the second reason for referral and the third identified diagnosis among all outpatient referrals and inpatient consultations. This is in agreement with a national survey conducted in Saudi Arabia, wherein moderate-to-severe symptoms of depression, anxiety, and stress were experienced in 28.3%, 24%, and 22.3% of the population during the pandemic [6].

Our sample showed that during lockdown, the number of patients referred because of substance and alcohol use disorders increased compared to the same period in 2019. This is in line with the American Medical Association's alarming data about a rising rate of drug relapse, overdose, and overdose deaths during the COVID-19 pandemic [12]. Regarding suicidality, the number of patients referred because of suicidal behavior was lower during the pre-lockdown, lockdown, and post-lockdown periods in 2020 compared to the previous year. This observation is similar to a time-series analysis of suicide rates in 21 countries where suicide numbers have remained constant with a slight decrease in the initial months of the pandemic [13]. On the contrary, a study in Malta showed a significant increase in suicidal behaviors (p<0.005) during the COVID-19 pandemic [14]. In Japan, a study showed a decrease in suicide rate early in the pandemic, followed by an increase in the second wave [15]. Suicide trends may fluctuate over time as the pandemic's long-term implications on health and the economy are still unclear [16].

Among all patients, a total of 420 patients failed to show up to their scheduled appointment. The increase in no-show rate in outpatient visits was statistically significant (p<0.05) in 2020. Similarly, a study in Italy showed a reduction in the number of mental health visits during lockdown with a decrease in the follow-up adherence (17.53%) compared to 30% in the previous year [17]. Moreover, Stewart et al. observed a reduction in mental health services and face-to-face contact during lockdown in the United Kingdom [18]. The transfer rate to the mental health hospital for admission has decreased slightly compared to the previous year. Our findings align with multiple studies conducted in the United Kingdom [8], Denmark [19], Italy [20], Germany [21], and Malta [15], where decreased overall psychiatric hospital admission was observed.

The present study has some limitations. This study was conducted in a single health care institute and the results cannot be generalized to all health care centers or regions. However, this study is the first in Saudi Arabia to have assessed the impact of the COVID-19 lockdown on psychiatric services utilization. Unfortunately, the lack of admission, addiction services in the study center, and the difficulty to follow up the patients who were referred to mental health centers may underestimate the overall utilization of psychiatric services. In Saudi Arabia, a single COVID-19 infection wave was reported, and one lockdown protocol was implemented. Therefore, our results provide limited information regarding the chronic and recurrent impact of COVID-19 waves and lockdowns. Nonetheless, our study provides essential observational points that could have implications for the response to future pandemics.

## Conclusions

This study provides insight into the impact of the COVID-19 pandemic on psychiatric services utilization. Our findings indicate that psychiatric services utilizations significantly reduced due to the COVID-19 lockdown. Mental health is a part of the health care system's response to public health emergencies. Promoting mental health services, identifying highly vulnerable individuals, and ensuring easy access to mental health resources are advised. Implementing evidence-based policy and protocol to guide mental health challenges in future health emergencies is needed. The present study's findings indicate the need for the application of telemedicine during the lockdown to ensure the availability of health services for individuals and populations that require them. The use of virtual clinics and telemedicine during the COVID-19 lockdown for supporting patients with health problems and psychiatric disorders has increased with a high acceptance rate from physicians and patients. This can be coupled with increasing the awareness of the psychological impact of the pandemic and the reinforcement of maintaining good mental health, in addition to informing the populations of the symptoms that need evaluation and the ways to seek primary and secondary health care services.
